# Knowledge of Health Services Access Among Hajj Pilgrims in Saudi Arabia During the 1445 H (2024 G) Season and Its Associated Demographic and Health-Related Factors

**DOI:** 10.3390/ijerph22101472

**Published:** 2025-09-24

**Authors:** Ghadah Sulaiman Alsaleh, Fahad A. Alamri, Jumanah Alhazmi, Lamis Alabdullatif, Faisal Fallatah, Mariyyah Alburayh, Anas Khan

**Affiliations:** 1 The Global Centre for Mass Gatherings Medicine, Ministry of Health, Riyadh 12382, Saudi Arabia; faabalamri@moh.gov.sa (F.A.A.); juaalhazmi@moh.gov.sa (J.A.); lalabdullatif@moh.gov.sa (L.A.); malburayh@moh.gov.sa (M.A.); or anaskhan@ksu.edu.sa (A.K.); 2 Ibn Sina National College for Medical Studies Jeddah, Al Mahjar, Jeddah 22421, Saudi Arabia; v.faisal@aol.com; 3 Department of Emergency Medicine, College of Medicine, King Saud University, Riyadh 12372, Saudi Arabia

**Keywords:** health services knowledge, Hajj pilgrims, demographic factors, health-related factors, health awareness, 1445 H (2024 G)

## Abstract

Background: The Hajj pilgrimage, a significant religious event, presents unique health challenges due to the large number of participants and the physical demands of the pilgrimage. Despite efforts by Saudi health authorities to provide comprehensive health services, the effectiveness of these interventions relies heavily on pilgrims’ knowledge of available services and emergency measures. Objectives: The aim of this study is to assess the level of knowledge of health services among Hajj pilgrims in Saudi Arabia during the 1445 H (2024 G) season and its associated demographic and health-related factors. Methods and Materials: This cross-sectional study was conducted during the 1445 H (2024 G) Hajj season with 1215 pilgrims, 51.7% male. Participants were randomly selected from selected pilgrimage accommodations or passing through the airport in Jeddah. Data were collected through face-to-face interviews using a structured questionnaire covering demographics, health characteristics, and knowledge of health services. Results: The study found that 62.1% of participants correctly believed that health services during Hajj were free of charge. Furthermore, 44.4% were aware of the emergency health number, with 31.5% exhibiting good knowledge. Bivariate analysis showed that younger participants had better knowledge (*p* < 0.001), and males were more likely than females to demonstrate partial or good knowledge (*p* = 0.011). Participants with university education were significantly more likely to have good knowledge, with 29.6% demonstrating good knowledge compared to 7.4% of illiterate participants (*p* < 0.001). Geographic region was also a factor, with 48.9% of Middle Eastern participants exhibiting good knowledge, compared to 27.1% of African and 23.6% of Asian participants (*p* < 0.001). However, multivariable logistic regression, revealed that only younger age (OR = 0.98, *p* = 0.005), university education (OR = 1.96, *p* = 0.024), and being from the Middle East (OR = 1.61, *p* = 0.009) were significant predictors of good knowledge of health services. Conclusions: The study identified significant gaps in pilgrims’ knowledge of health services during Hajj, with younger age, higher education, and Middle Eastern geographic region identified as independent predictors of better knowledge. These findings suggest the need for targeted health education initiatives, particularly for older pilgrims, those with lower educational attainment, and individuals from regions with lower awareness levels, to improve knowledge and potentially enhance health outcomes during Hajj.

## 1. Introduction

The Hajj pilgrimage, one of the five pillars of Islam, is an extraordinary religious event that attracts millions of Muslims from all corners of the globe to the Kingdom of Saudi Arabia each year [1]. It is a deeply significant spiritual journey, but it also presents substantial logistical and health-related challenges due to the sheer volume of participants and the physical demands of the pilgrimage rituals [2]. On average, around 2.5 million people participate in Hajj each year, making it one of the largest annual gatherings worldwide [3,4]. Given its global significance, the Hajj pilgrimage draws individuals from diverse backgrounds, cultures, and healthcare systems, complicating the delivery of effective health services [5]. This immense congregation in a limited geographic area poses unique public health challenges, including the prevention and management of infectious diseases, provision of emergency medical care, and education of pilgrims on health matters [6,7]. These challenges are further intensified by environmental factors, such as extreme weather conditions, and the physical demands of the pilgrimage rituals, which can exacerbate existing health conditions [8,9,10]. Ensuring the health and safety of pilgrims has thus become a priority for Saudi health authorities, who provide a range of services to address the complex needs of this population [11].

The Kingdom of Saudi Arabia, through its Ministry of Health and other relevant agencies, has made significant strides in developing a comprehensive healthcare system to support pilgrims during Hajj. This includes establishing healthcare facilities, deploying medical teams, and organizing preventative health campaigns to address issues like dehydration, heat stress, infectious diseases, and the challenges posed by pre-existing conditions such as hypertension and diabetes [5,12,13]. However, the effectiveness of these efforts is directly influenced by the level of knowledge pilgrims possess regarding the available health services. Health education plays a pivotal role in enabling pilgrims to make informed decisions about their health during the pilgrimage. By improving health literacy, pilgrims are better equipped to understand the importance of early intervention and preventive measures to reduce health risks [14].

Health-related knowledge among Hajj pilgrims is crucial because it directly influences their ability to access and utilize medical services when needed. A well-informed pilgrim can effectively navigate healthcare facilities, seek medical attention for common ailments or emergencies, and adhere to preventive health guidance such as vaccinations and hygiene practices. Conversely, a lack of knowledge can lead to delays in seeking medical care, poor health outcomes, or preventable health incidents [15,16]. This knowledge gap can also affect pilgrims’ understanding of their rights and responsibilities within the healthcare system, influencing their ability to access timely treatments and make informed decisions in emergencies [17]. Several studies have explored health awareness during the Hajj, but few have provided a comprehensive assessment of pilgrims’ overall understanding of health services, including emergency medical care, routine preventative measures, and available resources [18,19,20,21]. This highlights a gap in understanding how demographic and health-related factors collectively influence health service knowledge among pilgrims. Thus, research on the extent of pilgrims’ awareness remains limited, creating a critical gap in understanding how knowledge disparities may impact health outcomes.

The aim of this study is to assess Hajj pilgrims’ knowledge of health services during the year 1445 H (2024 G) and to explore the demographic and health-related factors associated with higher levels of this knowledge. By identifying these factors, the study aims to highlight areas where targeted health education interventions can be implemented, ultimately improving the health experience for pilgrims during Hajj.

## 2. Methods and Materials

### 2.1. Study Design and Participants

This was a cross-sectional study conducted during the 1445 H (2024 G) Hajj season. The study aimed to assess the level of knowledge of health services among Hajj pilgrims in Saudi Arabia during the 1445 H (2024 G) season and its associated demographic and health-related factors. Due to the challenging and dynamic environment of the Hajj, a pure random sampling method was not feasible. Instead, we employed a quota sampling strategy to ensure a diverse representation of the pilgrim population. Participants were recruited from selected pilgrimage accommodations and the arrival terminal at King Abdulaziz International Airport in Jeddah. Within these sites, trained data collectors approached pilgrims at different times of the day and in different locations (e.g., lobbies, common areas) to maximize variety. Quotas were set to roughly balance gender (male/female) and to include pilgrims from major geographic regions (Africa, Asia, Middle East, Europe). This sampling frame provided access to a large and diverse subset of the pilgrim population, though it did not include those arriving via all possible routes or accommodation types. A total of 1215 participants (628 males and 587 females) were included in the study. The study sought to include participants of both genders and from diverse ethnic backgrounds and age groups (all participants were ≥18 years).

### 2.2. Sample Size

The sample size for this study was determined using Epi Info software version 28. With a 95% confidence interval, a 50% prevalence, and a 3% margin of error, the calculated targeted sample size was 1066 participants. After adjusting for a projected 10% non-response rate, the final estimated sample size was at least 1185 participants. The study successfully recruited a total of 1215 participants, which exceeded the required sample size.

### 2.3. Inclusion and Exclusion Criteria

Participants were eligible for inclusion if they were male or female pilgrims aged 18 years or older who provided verbal informed consent and were able to complete the interview in either Arabic or English. There were no additional restrictions based on health status, geographic region, or ethnicity, ensuring a representative sample of pilgrims from diverse backgrounds. This broad inclusion criterion aimed to reflect the general health knowledge and experiences of the pilgrimage population.

### 2.4. Procedure and Data Collection

Data were collected using structured face-to-face interviews conducted by trained staff. No financial incentives or compensation were offered to ensure voluntary participation. A comprehensive questionnaire in both English and Arabic was used to collect relevant information from participants at their accommodations or airport facilities. Due to logistical constraints and to ensure the validity and consistency of the data collected, the study was limited to pilgrims who could complete the interview in either Arabic or English. The questionnaire was structured into two main sections, each focusing on specific aspects of the participants’ demographics and health knowledge.

The first section focused on collecting demographic and health characteristics of the study participants. This included variables such as age, gender, educational level, geographic region, the presence of chronic diseases, and whether participants had complained of any health symptoms during Hajj. Educational level categories included: illiterate (no formal education), ‘read and write’ (basic literacy skills without formal secondary education), intermediate or secondary, university, and not applicable (e.g., for participants who did not wish to disclose this information). Geographic region was classified into African, Asian, Middle Eastern, and European, while the presence of chronic diseases was recorded as “No” or “Yes”, and complaints of health symptoms were categorized as either “No” or “Yes”.

The second section assessed participants’ knowledge of health services and emergency numbers during Hajj. This section explored participants’ awareness of whether health services during Hajj were provided free of charge or required payment. The responses were categorized as “Free of charge”, “Paid”, “Don’t know”, or “N/A (not applicable/declined to answer)”. The ‘N/A’ category was used for respondents who did not provide a response to the question or were unable to answer.

Additionally, participants’ knowledge of emergency health numbers was assessed, with options of “No” or “Yes.” A composite “overall knowledge level” variable was then created based on performance in these two key questions (knowledge of service cost and knowledge of the emergency number). It was categorized as follows:

Good: Correctly answered both questions.
Partial: Correctly answered only one of the two questions.
No: Did not correctly answer either question.
N/A (not applicable): Participants with a non-response (‘N/A’) for both questions.

### 2.5. Ethical Considerations

This study was conducted in accordance with the ethical principles outlined in the Declaration of Helsinki, which ensures the protection of the rights, safety, and well-being of participants involved in clinical research. Ethical approval was granted by the Research Ethics Committee at King Fahad Medical City with the approval documented under IRB log Number: 24-292E. The confidentiality of participants’ personal information was maintained, and all data were anonymized to protect privacy. Additionally, the data were stored electronically on password-protected computers in a secure office. Access was restricted and limited solely to the principal investigator (PI) to ensure confidentiality and data integrity.

### 2.6. Statistical Analysis

Statistical analysis was conducted using SPSS software, version 28 (IBM Corp., Armonk, NY, USA). Numerical data were presented as mean ± standard deviation (SD) and analyzed using one-way ANOVA. Categorical data were presented as frequencies and percentages and analyzed using the Pearson Chi-square test or Fisher’s exact test, as appropriate. Univariate and multivariable logistic regression analyses were performed to identify factors associated with good knowledge. A two-tailed *p*-value of <0.05 was considered statistically significant.

## 3. Results

The demographic and health characteristics of the study participants are presented in Table 1. A total of 1215 individuals (628 males and 587 females) were included in the study, with a mean age of 52.44 ± 14.41 years. Regarding educational level, 29.9% had attended university, while 24.1% were literate. Over one-third of the participants (37.6%) were Middle Eastern, 31.6% were African, and 30.7% were Asian. Additionally, chronic diseases were reported by 36.4% of participants, and 58.5% reported experiencing health symptoms during Hajj.

Knowledge of health services and emergency numbers during Hajj among study participants are presented in Table 2. Most participants (62.1%) correctly believed that health services during Hajj were free of charge, while only 2.6% thought they were paid. Furthermore, 44.4% were aware of the emergency number to contact in case of a health issue, with 41.2% demonstrating partial knowledge and 31.5% exhibiting good knowledge.

The association of demographic and health factors with the knowledge level of health services during Hajj is presented in Table 3. Age showed a significant relationship with knowledge level, with younger participants exhibiting better knowledge (*p* < 0.001). Gender differences were also notable, as males were more likely than females to demonstrate partial or good knowledge (*p* = 0.011). Educational level was significantly associated with knowledge level, with university-educated participants having the highest proportion of good knowledge, while illiterate individuals had the lowest (*p* < 0.001). Geographic region also influenced knowledge levels, with Middle Eastern participants showing the highest percentage of good knowledge, followed by Africans and Asians (*p* < 0.001). Additionally, participants without chronic diseases and those not experiencing health symptoms during Hajj displayed better knowledge levels compared to their counterparts (*p* = 0.034 and *p* = 0.004, respectively).

The univariate and multivariable logistic regression analyses of factors associated with good knowledge of health services during Hajj are presented in Table 4. The univariate analysis identified age, education level, and geographic region as significant factors influencing knowledge levels. Younger age was associated with better knowledge, a relationship that persisted in the multivariable analysis (OR = 0.98, *p* = 0.005). University education emerged as a strong predictor of good knowledge in the multivariable model, with participants nearly twice as likely to have good knowledge compared to illiterate participants (OR = 1.96, *p* = 0.024). Additionally, Middle Eastern participants had significantly higher odds of good knowledge compared to African participants (OR = 1.61, *p* = 0.009). Other factors, including gender, chronic disease status, and health complaints during Hajj, were not significant predictors in the adjusted model.

## 4. Discussion

This study aimed to assess Hajj pilgrims’ knowledge of health services during the year 1445 H (2024 G) and to explore the demographic and health-related factors associated with higher levels of this knowledge. The findings revealed significant variations in knowledge levels, particularly based on age, educational background, and geographic region. These findings provide valuable insights into the effectiveness of health education during Hajj and offer a platform for future interventions.

One of the primary findings of this study was that 62.1% of pilgrims correctly believed that health services during Hajj are free of charge. This high percentage underscores the effectiveness of information dissemination by Saudi health authorities. The Saudi government offers free healthcare services to all pilgrims during Hajj, operating numerous hospitals and health centers to cater to their needs [22]. However, the fact that nearly a third of pilgrims were unsure or held misconceptions suggests that continued education and communication efforts are essential to ensure all pilgrims are well-informed.

Our study’s finding that only 44.4% of participants were aware of the emergency health number points to a critical gap in preparedness. This aligns with previous research indicating a lack of public awareness regarding emergency medical services (EMS) in Saudi Arabia. A study conducted in Riyadh by Alabdali et al. found that only 38.5% of the general public could recall the EMS phone number correctly [23]. This phenomenon likely stems from several interconnected factors. Firstly, information on emergency numbers may not be disseminated effectively through pre-travel briefings or prominently displayed in pilgrim accommodations. Secondly, educational efforts may be insufficiently tailored to overcome language barriers, as a single number (like 937) might be difficult to recall for non-Arabic speakers amidst a vast amount of new information. Finally, cultural barriers may play a role; pilgrims from regions with less developed EMS systems may not be accustomed to the concept of a universal emergency number, or may initially seek help from their group leaders rather than official channels. Therefore, the effectiveness of initiatives like the 937 Call Center in increasing awareness among specific groups, such as Hajj pilgrims, requires further evaluation and targeted promotion [24]. Future campaigns should ensure multi-lingual, repetitive, and context-specific messaging about the emergency number across all pilgrim touchpoints.

Demographic factors were strongly associated with knowledge levels. Our study found that younger pilgrims exhibited better knowledge of health services than older pilgrims (*p* < 0.001). This finding is consistent with generational differences in access to and familiarity with modern health communication tools, such as digital health resources [25,26]. This suggests that age-specific approaches to disseminating practical health service information are needed to bridge these gaps.

Educational level was another factor significantly associated with knowledge, with university-educated pilgrims showing the highest levels of awareness (*p* < 0.001). This finding reflects the general trend that higher levels of education correlate with better access to information and greater capacity to understand complex health-related topics. In line with our findings, a study by Aljahany et al. reported that individuals with higher education levels in Saudi Arabia had a significantly higher level of public health literacy [27]. Similarly, Jansen et al. reported that higher education attainment was associated with higher scores on the health literacy aspects appraisal of health information, and navigating the healthcare system. This association between education and knowledge likely reflects the better cognitive skills and resource access that come with higher education [28,29]. University-educated individuals are also more likely to engage with diverse sources of information, making them better equipped to understand and recall health-related information. This suggests that education-focused public health campaigns could improve awareness, but a tailored approach for less-educated pilgrims would be beneficial.

In terms of geographic region, Middle Eastern pilgrims were more knowledgeable about health services compared to African and Asian pilgrims (*p* < 0.001). This disparity could be attributed to differences in exposure to health education, healthcare systems, and cultural familiarity. This finding is supported by several studies; for instance, a study assessing the knowledge, attitude, and practices of pilgrims regarding heat-related illnesses during the 2017 Hajj found that pilgrims from the Middle East demonstrated better knowledge and attitude compared to those from Africa and Asia [30]. Another study assessing Muslim pilgrims’ knowledge, attitudes, and practices regarding complementary and alternative medicine (CAM) during Hajj season reported that a positive correlation was observed between geographic region and CAM self-practices [31]. The variation observed in our study could be explained by the differences in access to pre-travel information and the availability of Hajj-specific health campaigns in different regions. Middle Eastern pilgrims, being closer geographically and culturally to Saudi Arabia, may have had more exposure to health-related campaigns and services before their pilgrimage. On the other hand, African and Asian pilgrims might face barriers such as language differences and limited access to health resources, which can affect their awareness. This highlights the importance of tailoring health education campaigns to the diverse cultural and linguistic needs of pilgrims to ensure effective outreach.

It is important to note the distinction between initial associations and independent predictors. In our bivariate analysis, male gender, the absence of chronic diseases, and the absence of health symptoms during Hajj were also associated with higher knowledge levels. However, these associations were not sustained in the multivariable logistic regression model after adjusting for age, education, and region. This suggests that the apparent effects of gender, chronic disease status, and recent health symptoms are likely explained by confounding with the stronger, independent predictors identified in the final model (i.e., age, education, and geographic region). The observed bivariate gender difference, for instance, may be attributable to broader structural factors, such as disparities in access to education or culturally defined social roles that vary across regions, which are captured by the education and geographic variables in the model. Therefore, the discussion of core findings will focus on these three key predictors.

The multivariable analysis in this study highlights younger age, higher education, and Middle Eastern geographic region as significant predictors of better knowledge of health services among Hajj pilgrims (*p* < 0.05). These findings emphasize structural and behavioral factors that shape health literacy and access to information, with important implications for public health interventions. The association between younger age and better knowledge may reflect generational differences in access to digital and social media-based health communication. Younger individuals are more likely to use online platforms, smartphone applications, and interactive health resources, making them better informed about available services. This suggests that digital health strategies, including mobile health (mHealth) applications and online pre-Hajj educational modules, could be leveraged to reach a broader audience, particularly older pilgrims who may be less familiar with such resources. Similarly, the strong correlation between higher education and increased knowledge levels reinforces the role of knowledge of available services in shaping healthcare utilization. Education enhances an individual’s ability to seek, interpret, and apply health information, allowing for better navigation of complex healthcare systems. While higher-educated pilgrims may naturally engage with health-related materials, lower-educated pilgrims may require simplified, multilingual, and culturally adapted educational materials to bridge knowledge gaps. Health authorities should consider expanding pre-departure orientation sessions and using visual or audiovisual content to enhance knowledge retention among less-educated pilgrims. The influence of Middle Eastern geographic region as a predictor of good knowledge suggests that geographic and cultural proximity to Saudi Arabia may play a role in shaping pilgrims’ awareness of health services. Middle Eastern pilgrims might have prior exposure to Hajj-related health campaigns, easier access to Arabic-language materials, or familiarity with the Saudi healthcare system, giving them a knowledge advantage. In contrast, non-Middle Eastern pilgrims, particularly those from African and Asian countries, may face linguistic, systemic, and infrastructural barriers that limit their access to health information. Strengthening collaborations with sending countries to standardize and expand pre-travel health education programs can help ensure that all pilgrims, regardless of geographic region, receive equal access to critical health information.

## 5. Strengths and Limitations

This study can be evaluated in terms of its strengths and limitations. One of the key strengths is the large sample size of 1215 participants, which significantly enhances the statistical power and generalizability of the findings to the broader population of Hajj pilgrims. The study’s diverse participant pool, representing various ethnic backgrounds, genders, and age groups, strengthens the external validity by minimizing selection bias. The comprehensive data collection through structured questionnaires enabled an in-depth assessment of both demographic and health-related factors, allowing for a detailed analysis of pilgrims’ knowledge of health services. Additionally, the use of multivariable logistic regression identified key predictors of good knowledge, while controlling for potential confounders.

Ethical rigor was maintained throughout the study, with strict measures ensuring participant confidentiality and data integrity. However, the study has some limitations. The cross-sectional design limits the ability to infer causal relationships, as the findings reflect a snapshot in time. The reliance on self-reported data could also introduce bias, as participants may overestimate their knowledge or underreport certain health conditions. Furthermore, the study’s geographic scope was limited to Pilgrims who were either staying at selected pilgrimage accommodations or passing through the airport in Jeddah, which may not fully represent the experiences of pilgrims in more remote areas. Furthermore, while quota sampling was employed within the selected sites, participants were recruited only from specific accommodations and the Jeddah airport. This approach may have excluded pilgrims arriving through other entry points (e.g., Madinah airport or land borders) or those staying in non-commercial or government-provided housing, potentially limiting the generalizability of our results. Furthermore, while quotas were used to encourage diversity, the use of a non-probability quota sampling method means that our sample may not be fully representative of the entire Hajj population, and the results may be subject to selection bias. The findings should be interpreted with this caveat in mind. Future studies should aim for a more geographically dispersed sampling frame and could employ direct standardization techniques for key demographic variables (e.g., age and sex) to better represent the entire Hajj population and improve the accuracy of prevalence estimates. Additionally, the requirement for participants to be proficient in either Arabic or English to provide informed consent and complete the interview may have excluded non-speaking pilgrims, potentially limiting the representativeness of our findings for that demographic group. This is an important consideration as knowledge levels may differ among pilgrims who do not speak these languages. This language barrier means our findings cannot be generalized to pilgrims who do not speak these languages, who may have systematically different levels of knowledge or access to information. Furthermore, it is important to note that our study measured a specific facet of knowledge, awareness of service cost and the emergency number, as a proxy for understanding how to access care. This is a narrow, though practical, component of the broader and more multidimensional concept of health literacy, which encompasses functional, communicative, and critical skills to navigate the healthcare system. Therefore, our findings should not be interpreted as representing pilgrims’ overall health literacy. Future research could employ comprehensive health literacy tools to gain a more holistic understanding. Lastly, the absence of a longitudinal follow-up means that the study could not assess whether improvements in health service knowledge would be sustained beyond the Hajj season, suggesting the need for future research that includes longitudinal data.

## 6. Clinical and Practical Implications

This research is significant for several reasons. First, it provides a comprehensive and updated understanding of the level of health services knowledge among Hajj pilgrims, which is essential for improving healthcare delivery during the pilgrimage. Second, by identifying demographic and health-related factors that contribute to knowledge gaps, this study offers valuable insights that can inform the design of targeted health education campaigns tailored to the needs of specific groups of pilgrims. For example, health services may need to focus more on educating elderly pilgrims or those with limited education about how to access medical care and adhere to preventative health measures. Third, the findings of this study have broader implications for public health policies, both within the context of the Hajj and for large-scale religious or mass gatherings globally. Understanding the knowledge gaps in such environments is crucial for improving public health outcomes and preventing avoidable health crises.

The implications of this study extend beyond the immediate context of the Hajj pilgrimage. The insights gained can help refine health communication strategies and health education frameworks for other mass gatherings, where the combination of a large, diverse, and often transient population presents unique public health challenges. The study’s findings will not only help optimize health services during Hajj but also contribute to the broader field of public health by providing a model for how to assess and address health literacy gaps in large-scale events. Ultimately, this research will play a pivotal role in ensuring that health services during Hajj are as effective and accessible as possible, enhancing the safety and well-being of pilgrims and contributing to the global understanding of health management at mass gatherings.

## 7. Conclusions

This study provides important insights into the knowledge of health services among Hajj pilgrims and identifies key demographic factors influencing this knowledge. The findings suggest that pre-arrival educational interventions tailored to specific groups, particularly older pilgrims, those with lower educational attainment, and individuals from regions with less access to health information, could significantly improve health outcomes during Hajj. The results contribute to a growing body of literature on health service knowledge during mass gatherings and offer practical implications for enhancing public health education in future Hajj seasons.

## 8. Strategic Recommendations

To address the disparities in knowledge levels identified in this study, a multi-faceted approach is needed to enhance health awareness among all Hajj pilgrims. First, expanding digital health communication strategies can be an effective way to reach a broad audience, particularly younger individuals who are more accustomed to using technology for health information. Developing multilingual and culturally tailored mobile health (mHealth) applications, sending targeted SMS alerts, and leveraging social media platforms can ensure that critical health service information is disseminated efficiently before and during Hajj. Additionally, incorporating interactive elements such as virtual tutorials or chatbot-assisted guidance in multiple languages could further enhance engagement and retention of information.

Second, strengthening pre-departure training programs is essential to ensure that all pilgrims, regardless of geographic region, receive uniform and standardized health education. Collaborating with health authorities in pilgrims’ home countries to implement comprehensive pre-Hajj health education initiatives can help bridge knowledge gaps, particularly for non-Middle Eastern pilgrims who may have limited prior exposure to Saudi Arabia’s healthcare system. These programs should emphasize key health services, emergency procedures, and preventive measures, with content adapted to the linguistic and cultural needs of different populations.

Moreover, simplifying health education materials is crucial to ensure accessibility for individuals with lower educational backgrounds. The use of pictograms, short videos, and infographics can effectively communicate complex health information in a more digestible format. Providing translated materials in widely spoken languages such as Urdu, Bengali, Hausa, and Bahasa Indonesia can further facilitate understanding among non-Arabic-speaking pilgrims. Ensuring that these resources are readily available in travel agencies, mosques, and community centers before departure can maximize their impact.

Lastly, improving accessibility to health information for older pilgrims should be a key priority. Many older individuals may face barriers related to digital literacy, limiting their ability to access online health resources. Deploying trained community health workers or volunteers at key points, such as Hajj terminals, hotels, and health centers, can provide on-the-ground assistance to help older pilgrims navigate healthcare services. Additionally, offering in-person health briefings at gathering points in Saudi Arabia could serve as a reinforcement mechanism for those who may have missed or not fully comprehended pre-departure education.

By implementing these strategic interventions, health authorities can work towards ensuring equitable access to health information for all Hajj pilgrims, thereby enhancing their preparedness, reducing healthcare burdens, and ultimately improving health outcomes during the pilgrimage.

## Figures and Tables

**Table 1:** Demographic and Health Characteristics of the Study Participants (*n* = 1215).

Item	*n*	%
Age (years), Mean (SD)	52.44 (14.41)	
Gender		
Male	628	51.7
Female	587	48.3
Educational level		
Illiterate	114	9.4
Read and write	293	24.1
Intermediate or secondary	132	10.9
University	363	29.9
N/A	313	25.8
Geographic Region		
African	384	31.6
Asian	373	30.7
Middle eastern	457	37.6
European	1	0.1
Chronic diseases		
No	773	63.6
Yes	442	36.4
Complaining of health symptoms during Hajj		
No	504	41.5
Yes	711	58.5

**Table 2:** Knowledge of Health Services and Emergency Numbers During Hajj Among Study Participants (*n* = 1215).

**Knowledge Item**	***n***	**%**
Knowledge of health service fees		
Free of charge	755	62.1
Paid	32	2.6
I don’t know	376	30.9
N/A *	52	4.3
Knowledge of emergency number		
No	676	55.6
Yes	539	44.4
Overall knowledge level		
No	280	23.0
Partial	500	41.2
Good	383	31.5
N/A *	52	4.3

* N/A = not applicable.

**Table 3:** Association of Demographic and Health Factors with Knowledge Level of Health Services During Hajj.

		Knowledge Level		
Item	No (*n* = 280)	Partial (*n* = 500)	Good (*n* = 383)	*p*-Value
Age (years)	56.75 ± 15.35	52.2 ± 13.46	49.63 ± 13.87	<0.001
Gender				
Male	122 (20.5%)	272 (45.6%)	202 (33.9%)	0.011
Female	158 (27.9%)	228 (40.2%)	181 (31.9%)	
Educational level	(*n* = 235)	(*n* = 360)	(*n* = 261)	
Illiterate	61 (57%)	27 (25.2%)	19 (17.8%)	
Read and write	93 (33.0%)	109 (38.7%)	80 (28.4%)	<0.001
Intermediate or secondary	21 (16.9%)	68 (54.8%)	35 (28.2%)	
University	60 (17.5%)	156 (45.5%)	127 (37%)	
	(*n* = 280)	(*n* = 499)	(*n* = 383)	
Geographic Region				
African	87 (23.5%)	170 (45.8%)	114 (30.7%)	
Asian	123 (34.4%)	139 (38.8%)	96 (26.8%)	<0.001
Middle eastern	70 (16.2%)	190 (43.9%)	173 (40.0%)	
Chronic diseases				
No	190 (25.9%)	295 (40.2%)	248 (33.8%)	0.034
Yes	90 (20.9%)	205 (47.7%)	135 (31.4%)	
Complaining of health symptoms during Hajj				
No	129 (27.0%)	178 (37.3%)	170 (35.6%)	0.004
Yes	151 (22.0%)	322 (46.9%)	213 (31.0%)	

Numerical data are presented as mean (SD) and analyzed using One-way ANOVA while categorical data are presented as frequency (%) and analyzed using Pearson Chi square test, Statistical significance at *p* value < 0.05, participants with N/A knowledge were excluded from the analysis.

**Table 4:** Univariate and Multivariable Logistic Regression for Factors Associated with Good Knowledge of Health Services During Hajj.

Univariate					Multivariable			
Factors	OR	95% CI		*p*-Value	OR	95% CI		*p*-Value
		Lower	Upper			Lower	Upper	
Age	0.98	0.97	0.99	<0.001	0.98	0.97	1.001	0.005
Gender								
Female	1.00				1.00			
Male	1.09	0.86	1.4	0.475	1.15	0.85	1.56	0.363
Education level								
Illiterate	1.00				1.00			
Read and write	1.83	1.05	3.21	0.034	1.52	0.85	2.71	0.159
Intermediate or secondary	1.82	0.97	3.42	0.063	1.37	0.71	2.65	0.351
University	2.72	1.58	4.68	<0.001	1.96	1.09	3.51	0.024
Geographic Region								
African	1.00				1.00			
Asian	0.83	0.6	1.14	0.244	1.09	0.73	1.63	0.663
Middle eastern	1.5	1.12	2.01	0.007	1.61	1.13	2.31	0.009
Having Chronic disease	0.89	0.69	1.15	0.393	1.00	0.71	1.42	0.985
Complaining of health symptoms during Hajj	0.81	0.63	1.04	0.102	1.02	0.75	1.40	0.894

## Data Availability

The dataset used and/or analyzed during the current study are available from the corresponding author on reasonable request.
